# Recombinant Tax1 and Tax2 proteins inhibit HIV-1 replication in peripheral blood mononuclear cells

**DOI:** 10.1186/1742-4690-11-S1-P136

**Published:** 2014-01-07

**Authors:** Christy S Barrios, Laura Castillo, Li Wu, Chou-Zen Giam, Mark A Beilke

**Affiliations:** 1 Department of Medicine and Veteran’s Affairs Research Service, Medical College of Wisconsin, Milwaukee, Wisconsin, USA; 2 Department of Veterinary Biosciences and Center for Retrovirus Research, The Ohio State University, Columbus, Ohio, USA; 3 Department of Microbiology and Immunology, Uniformed Services University of the Health Sciences, Bethesda, Maryland, USA

## 

Patients with HIV-1 and HTLV-2 coinfections often exhibit a clinical course similar to HIV-1 infected long-term nonprogressors. This observation has been attributed in part to the ability of the HTLV Tax2 protein to activate production of antiviral chemokines and to downregulate the CCR5 co receptor on lymphocytes. Therefore we investigated the possibility that a recombinant Tax2 protein could suppress HIV-1 viral replication *in vitro*. R5-tropic HIV-1 (NLAD8)-infected-PBMCs were treated daily with recombinant Tax1 and Tax2 proteins (dosage range 1-100 pM). Culture supernatants were collected at intervals for 22 days post-infection and assayed for levels of HIV-1 p24 antigen. Treatment of PBMCs with Tax2 protein resulted in significant reduction in HIV-1 p24 antigen levels (*p*<0.05) at days 10, 14, and 18 post-infection compared to HIV-1-infected or mock-treated PBMCs. This was preceded by the detection of increased levels of CC-chemokines MIP-1α/CCL3, MIP-1β/CCL4, and RANTES/CCL5, on days 1-7 of infection (*p*<0.05). Similar, but less robust inhibition was determined in Tax1 treated PBMCs. Addition of Tax2 proteins starting 48 hours previous to infection resulted in a significant inhibition of HIV-1 p24 levels (*p*<*0.*05 at days 7 to 14 compared to the untreated, R5 infected PBMCs. In contrast, when addition of Tax2 began two days after R5 infection, significant HIV-1 p24 levels were only determined at days 7 and 10 for 1 pM and at day 7 for 10 pM (*p*<*0.05*)*.* These results support the contention that Tax1 and Tax2 play a role in generating antiviral responses against HIV-1 *in vivo* and *in vitro*.

